# Hypoxia and the Receptor for Advanced Glycation End Products (RAGE) Signaling in Cancer

**DOI:** 10.3390/ijms22158153

**Published:** 2021-07-29

**Authors:** Sakshi Taneja, Stefan W. Vetter, Estelle Leclerc

**Affiliations:** Department of Pharmaceutical Sciences, North Dakota State University, Fargo, ND 58105, USA; sakshi.taneja@ndsu.edu (S.T.); stefan.vetter@ndsu.edu (S.W.V.)

**Keywords:** hypoxia, HIF-1α, RAGE, S100 proteins, HMGB1, cancer

## Abstract

Hypoxia is characterized by an inadequate supply of oxygen to tissues, and hypoxic regions are commonly found in solid tumors. The cellular response to hypoxic conditions is mediated through the activation of hypoxia-inducible factors (HIFs) that control the expression of a large number of target genes. Recent studies have shown that the receptor for advanced glycation end products (RAGE) participates in hypoxia-dependent cellular adaptation. We review recent evidence on the role of RAGE signaling in tumor biology under hypoxic conditions.

## 1. Physiological and Pathological Hypoxia

Hypoxia is a condition characterized by low levels of oxygen in tissues and organs. Two main types of hypoxia have been described: physiological and pathological hypoxia [1]. Physiological hypoxia is observed when oxygen levels are slightly lower than typical under healthy conditions and when these levels can be easily reversed by homeostatic mechanisms [2]. For instance, in peripheral tissues, where oxygen levels vary between about 3% to 7%, physiological hypoxia is observed at oxygen levels between 2% and 6% [1]. On the other hand, pathological hypoxia is characterized by oxygen levels significantly lower than those in healthy tissues and can reach levels of less than 1% oxygen saturation [1]. Pathological hypoxia develops because homeostatic mechanisms are not able to restore the supply of the tissue with oxygen to its physiological level [3]. Pathological hypoxia is observed in many solid tumors and has been shown to contribute to tumor growth, metastasis, and chemoresistance [1,4]. On the cellular level, hypoxia is sensed by transcription factors named hypoxia-inducible factors (HIF*s).* HIFs regulate the transcription of hundreds of genes, some of them critically involved in cancer processes contributing to tumorigenesis, chemoresistance, and metastasis [5,6].

## 2. Hypoxia-Inducible Factors: Structure and Mechanisms

The first HIF was discovered in 1991 by Semenza et al. as a protein interacting with a DNA response element present on the erythropoietin gene [7]. Since its initial discovery, two additional hypoxia-responsive members of the HIF family have been identified. Collectively, the HIFs regulate the transcription of a large number of genes involved in many aspects of cancer, such as metabolic reprogramming, cell proliferation and invasion, metastasis, and chemoresistance [5,8,9]. The functions of the three HIFs in the cellular response to hypoxia are complementary yet distinct [10,11].

### 2.1. HIF Subunits

HIFs function as heterodimers consisting of two subunits: an oxygen-sensitive α-subunit and an oxygen-independent β-subunit. The genes encoding the HIF-1α, HIF-2α (approved gene symbol EPAS1, endothelial PAS domain protein 1), and HIF-3α are located on chromosomes 14, 2, and 19, respectively. Two HIF β-subunits have been described and are known as the aryl hydrocarbon nuclear translocator ARNT1 (HIF-1β) and ARNT2 (HIF-2β) [12].

All three HIF-α subunits can form heterodimers with HIF-1β, bind to hypoxia response elements (HREs) of HIF-1α target genes, and modulate their expressions, resulting in cellular adaptation to hypoxia [5,7,13,14]. HIF-α forms are differentially expressed in tissues. At the RNA level, HIF-1α is found expressed in most tissues, whereas the expression of HIF-2α is limited mostly to vascular tissues (lung, heart, placenta, and kidney) [15,16,17]. HIF-3a mRNAs have been detected in adult thymus, lung, heart, and kidney [18,19,20]. Splice variants have been reported for HIF-1α and, in particular, for HIF-3α [21]. The expression of HIF-3a splice variants appears to be tissue-specific, with different variants being expressed at different levels in distinct tissues [22]. Interestingly, some splice variants of HIF-3α appear capable of binding HIF-1α and of suppressing HIF-1α activity [23].

### 2.2. Domain Organization

**Figure 1:**
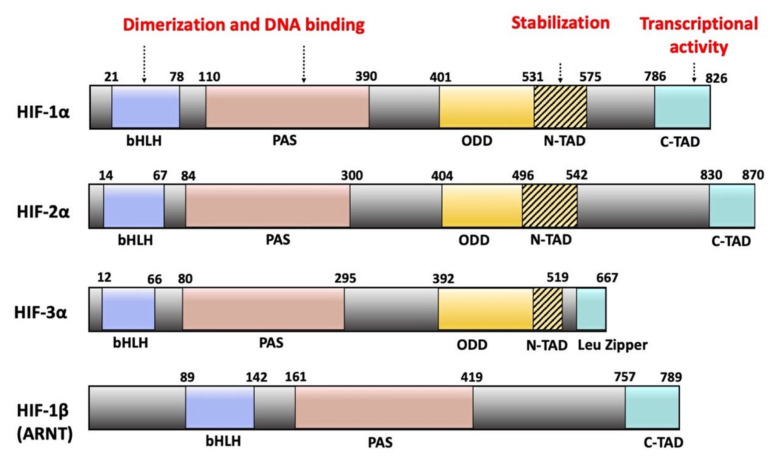
Domain architecture of HIF-α and HIF-1β subunits. All subunits contain bHLH and twin PAS domains. These domains contain the DNA binding and heterodimerization sites. All three HIF-α forms possess an oxygen-dependent degradation (ODD) domain. HIF-1α and HIF-2α possess two transactivation domains (N-TAD and C-TAD). N-TAD is responsible for stabilizing all three forms of HIF-α under hypoxic conditions, while C-TAD controls the transcription of HIF-α target genes. The N-TAD domains are part of the ODD domains. HIF-3α possesses a leucine zipper (LZIP) domain instead of a C-TAD domain. HIF-1β lacks both the ODD and N-TAD domains.

The HIF-1α and HIF-2α paralogs share an identical domain architecture and 48% sequence similarity [5,15,18]. HIF-3α differs from the other two HIF-αs in the C-terminal domain organization and is about 200 amino acids shorter in length [16,24] (Figure 1).

Structurally, all three HIF-α and the HIF-1β subunits contain a basic helix-loop-helix (bHLH) and a twin Per-ARNT-Sim (PAS) domain in the N-terminal part of the protein. These domains are responsible for heterodimerization between the α and β subunits (Figure 1) [25].

All three HIF-α forms are oxygen-sensitive due to the presence of an oxygen-dependent degradation (ODD) domain [26]. The ODD domains are defined through functional studies rather than by a conserved three-dimensional protein fold. In addition, HIF-1α and HIF-2α subunits contain two functional transactivation domains: the N-terminal transactivation domain (N-TAD) and the C-terminal transactivation domain (C-TAD). Functional studies have identified some sequence overlap between the ODD and N-TAD regions [27].

In association with transcriptional coactivators such as the coactivator binding protein (CBP) and the p300 protein, C-TAD controls the transcription of HIF-1α and HIF-2α target genes while N-TAD protects these α subunits against oxygen-dependent degradation [24,28]. In contrast, HIF-3α contains a leucine zipper (LZIP) domain instead of the C-TAD domain [12,29]. The LZIP domain participates in protein-protein interactions [30]. HIF-1β lacks both the ODD and N-TAD domains but possesses a C-TAD domain (Figure 1) [30].

### 2.3. Regulation Mechanisms

The HIFs themselves cannot sense oxygen directly and depend on cellular enzymes to introduce posttranslational modifications into HIF-α in an oxygen-dependent manner. Two posttranslational mechanisms of HIF-α modifications regulate the proteolytic degradation of HIF-α. The HIF-β subunits are not responsive to cellular oxygen concentrations.

The first mechanism is the oxygen-dependent hydroxylation of specific proline residues in the ODD region and adjacent to the N-TAD domain of HIF-α subunits. In HIF-1α and HIF-2a, two conserved proline residues (P402 and P564, and P405 and P531, respectively) are hydroxylated by proline-hydroxylases (PHDs) under physiological oxygen levels. These hydroxylated proline residues are recognized by the von Hippel-Lindau protein (pVHL), which then forms a larger complex with additional proteins such as elongin C, elongin B, cullin-2, and Rbx1, resulting in ubiquitination and subsequent proteosomal degradation of HIF-α [31,32,33]. In HIF-3a, only hydroxylation of P492 is necessary for recognition by pVHL [31]. Under hypoxic conditions, PHDs lose their activity and cannot hydroxylate proline residues, resulting in the stabilization of HIF-α subunits [12,33]. Subsequently, the HIF-α subunits translocate to the nucleus and heterodimerize with HIF-1β. The heterodimers then bind to HREs of target genes, resulting in the activation of a variety of signaling pathways [34]. Because the HIF-1β subunit does not contain an ODD domain, HIF-1β expression levels are not dependent on the presence of oxygen [5]. The interaction between HIF-1α and pVHL has also been shown to be enhanced following acetylation of Lysine residue 532 present in the ODD domain of HIF-1α through the activation of an oxygen-independent acetyltransferase (arrest-defective-1 (ARD1)) [35]. Besides being degraded through the pVHL-dependent pathway, HIF-1α can be ubiquitinated and later degraded through the activation of other pathways, including the oncogenic E3 ubiquitin ligase murine double minute 2 (MDM2) and the p53 protein [36].

The second main mechanism of oxygen-dependent regulation of HIF-1α activity involves the hydroxylation of a specific asparagine residue in the C-terminal TAD domain. Under normoxic conditions, hydroxylation of asparagine 803 in the C-TAD domain of HIF-1α by an asparaginyl hydroxylase enzyme named factor-inhibiting HIF1 (FIH) prevents the interaction between HIF-1α with transcriptional coactivators [37]. In order to be fully active, HIF-1α needs to form complexes through the N-TAD and C-TAD domains with transcriptional coactivators such as CREB binding proteins (CBP) and p300, SRC-1, and TIF2 [38,39,40,41]. At the physiological oxygen level, hydroxylation of Asp 803 prevents assembly with coactivators. Under hypoxic conditions, hydroxylation of asparagine 803 does not occur, thus resulting in the formation of active transcriptional complexes between the C-TAD of HIF-1α and the transcriptional coactivators [42,43].

HIF-1α can also be regulated through oxygen-independent mechanisms. One mechanism involves the interaction between HIF-1α and the hypoxia-associated factor (HAF), a specific E3 ubiquitin ligase, resulting in oxygen-independent proteosomal degradation of HIF-1α [44]. Interestingly, the interaction of HAF with HIF-2α does not result in the same outcome: HIF-2α does not become ubiquitinylated and is not degraded. Instead, the interaction between HAF and HIF-2α results in increased activity of HIF-2α [45].

In addition, HIFs have been found to be regulated through direct phosphorylation by the MAP kinases p38 and ERK1/2 or through the phosphorylation of coactivators of HIFs, in a cell type-dependent manner [46,47,48,49]. HIF-1α expression has also been shown to be regulated through the activation of growth factor receptors by their ligands (such as insulin or insulin-like growth factor 1) or other external stimuli through the activation of the PI3K pathway [50,51,52,53]. HIF-α can also be regulated directly through the activation of NF-kB, and binding sites for NF-kB have been identified in the promoter region of HIF-1α [54].

## 3. Effect of Hypoxia in Tumors

Many tumor tissues show higher expression levels of HIF-1α than normal tissues. For instance, immunohistochemical studies of tissues from bladder, breast, colon, glial, hepatocellular, ovarian, pancreatic, prostate, and renal tumors showed positive staining for either HIF-1α or HIF-2α or both, as compared to negatively stained control adjacent tissues, with the exception of bone marrow macrophages [49,50]. In these tissues, staining was found to be mostly nuclear [55,56]. The presence of hypoxic regions in tumors is explained in part by the fast-growing rate of tumors, where the demand for oxygen exceeds its supply, and also because of defective vascularization [57]. Indeed, the tumor vasculature is leaky and more fragile than the vasculature found in healthy tissues [58]. In response to hypoxia, a large cellular reprograming is initiated and coordinated by HIFs. Activation of HIFs has been shown to contribute to many cellular processes described as cancer “hallmarks”, including metabolic reprogramming, angiogenesis, apoptosis, and invasion and metastasis [1,4,59,60].

### 3.1. Metabolic Reprogramming

Under hypoxic conditions, cancer cells reprogram their metabolism by switching from oxygen-dependent mitochondrial oxidative phosphorylation to oxygen-independent glycolysis in order to meet their energy requirements; this metabolic switch is referred to as the Warburg effect [61]. This switch is controlled by the HIF-dependent upregulation of a large number of enzymes of the glycolytic pathway as well as glucose transporters (reviewed in the work of [5]).

### 3.2. Angiogenesis

Another critical response to hypoxia by cancer cells is angiogenesis. Angiogenesis is triggered by the upregulation of proangiogenic factors, including vascular endothelial growth factor (VEGF) (reviewed in the work of [62]). The formation of new blood vessels promotes tumor cell survival by increasing the delivery of nutrients and oxygen to tumor cells [63,64].

### 3.3. Apoptosis

The effect of hypoxia on apoptosis is complex and conflicting results have been observed where activation of HIF-1α resulted in either increased or decreased apoptosis [65,66,67]. The p53 protein is a transcription factor with critical roles in apoptosis [68]. Hypoxia has been shown to increase apoptosis in tumors through the accumulation of p53 [69]. HIF-1α has also been suggested to stabilize p53 through its direct interaction with the Mdm2 protein and by inhibiting Mdm2-mediated p53 degradation [70,71]. However, although hypoxia can lead to apoptosis and reduced cell proliferation, hypoxia correlates with tumor aggressiveness and is associated with poor patient prognosis in many cancer types [72]. To explain this paradoxical effect of hypoxia on apoptosis, it has been suggested that hypoxic conditions can serve as a selective pressure in growing tumors, favoring the elimination of wild-type p53 expressing cells and the growth of cells with inactive p53 [73]. Another study also suggested that rather than enhancing the transactivation properties of p53, hypoxia could lead to increases in p53 transrepression activities, resulting in a significant decrease in p53 dependent apoptosis [74]. The role of p53 in apoptosis is still open for investigation [68], and future studies will certainly enhance our understanding of its role in hypoxic conditions.

### 3.4. Cell Invasion and Metastasis

The roles of hypoxia and HIF-1α on tumor cell invasion and metastasis are well established [75]. It has been shown that intratumoral hypoxia is associated with metastasis (reviewed in the work of [60]). Under hypoxic conditions, cancer cells can undergo epithelial to mesenchymal transition (EMT) that is associated with higher cell motility and reduced cell-cell adhesion [76]. Hypoxia also increases cell invasion through the activation of matrix metalloproteases (MMPs) [77]. In addition, hypoxic conditions have been shown to support cancer stem-cell self-renewal through the activation of HIF-dependent activation of several genes, including OCT4 [78]. Under hypoxic conditions, the colonization of tumor cells at distant sites is enhanced through the upregulation of angiopoietin-like 4 (ANGPTL4) that disrupts contacts between endothelial cells and favors the passage of tumors cells through the blood vessels [76].

### 3.5. Tumor Microenvironment and Inflammation

Hypoxia has been linked to inflammation in the tumor microenvironment through the activity of HIFs as well through the activation of the master regulator of inflammation, NF-κB [79,80,81]. In the tumor, under hypoxic conditions, HIF-1α expression and activity are regulated by NF-κB [82,83]. The tumor microenvironment contains many types of non-cancer cells, including immune cells, and hypoxia has been shown to modulate the function of immune cells and to result in inflammation [84,85,86,87,88,89]. Importantly, the tumor microenvironment is rich in damaged-associated molecular patterns (DAMPs) or alarmins that further contribute to an inflamed tumor microenvironment [90,91]. Alarmins or DAMPs influence tumor growth through their activation with their receptors; one of these receptors is the receptor for advanced glycation end products (RAGE) [92,93,94].

## 4. The Receptor for Advanced Glycation End Products

The receptor for advanced glycation end products (RAGE) was first identified in endothelial cells of bovine lungs as a binding partner for advanced glycation end products (AGEs) [95]. RAGE belongs to the large immunoglobulin superfamily, and its gene is located in the class III major histocompatibility complex (MHC) region on chromosome 6 [96]. The presence of the RAGE gene within this complex suggests a critical role of RAGE in inflammation [97].

### 4.1. RAGE: Domain Structure and Isoforms

**Figure 2:**
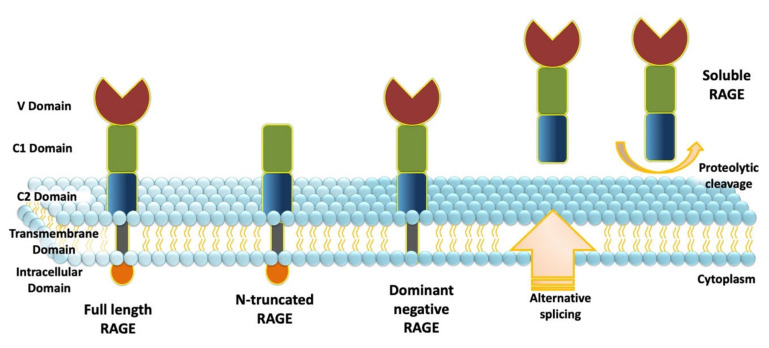
RAGE structure and isoforms. Full-length RAGE consists of three regions: an extracellular part itself divided into a variable-like (V), two constant-like Ig (C1 and C2) domains, a transmembrane domain, and a cytoplasmic domain. The soluble form of RAGE (sRAGE) contains the extracellular region but lacks both the transmembrane and cytoplasmic domains. The dominant negative isoform lacks the cytoplasmic domain, while N-truncated RAGE is deficient in the N-terminal V-like domain.

The full-length form of RAGE consists of three main regions: an extracellular part (amino acids 23–342) itself divided into a variable-like (V) and two constant-like (C1 and C2) immunoglobulin (Ig) domains, a transmembrane domain (amino acids 343–363), and a cytoplasmic domain (amino acids 364–404) (Figure 2) [95]. Two other isoforms of RAGE are the N-truncated RAGE (amino acids 124–404), where the N-terminal V-like domain is deleted [98], and the dominant negative isoform (amino acids 23–363), where the cytoplasmic domain is missing. In addition, two different types of soluble isoforms of RAGE (sRAGE) (amino acids 23–342) have been described [99]. sRAGE contains the extracellular part but lacks the transmembrane and cytoplasmic domains. sRAGE can be generated as a result of a splicing event or by proteolytic cleavage of full-length RAGE [100].

### 4.2. RAGE Signaling Pathways in Cancer

RAGE signaling contributes to many physiological and pathophysiological processes and diseases such as innate immune responses, tissue repair, bone homeostasis, complications of diabetes, Alzheimer’s disease, as well as cancers (brain, breast, colon, colorectal, prostate, pancreatic oral squamous cell, ovarian cancer, and melanoma) [101,102,103,104,105,106,107,108,109]. In general, RAGE signaling is initiated by the binding of its ligands to the extracellular part of the receptor. The outcome of this interaction depends on the type of ligand, its concentration, as well as the cell type (reviewed in the work of [110]). Upon RAGE engagement by its ligand, the cytoplasmic domain of RAGE transmits signals with the help of adaptor proteins [111,112,113]. Three adaptor proteins have been identified: diaphanous-1 (Dia-1), TIRAP, and MyD88. Dia-1 has been shown to play an important role in RAGE-dependent cell migration through the activation of Rac1 and Cdc42 [114]. TIRAP and MyD88 also function as adaptors to toll-like receptors (TLR) 2 and 4 [115].

**Figure 3:**
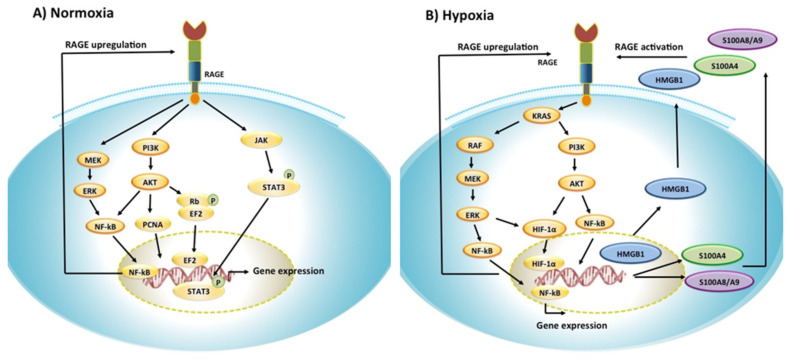
RAGE signaling pathways in normoxia and hypoxia. (**A**) In normoxia, RAGE activation by its ligands results in the activation of the MEK/ERK, PI3K/Akt, and JAK/STAT pathways, resulting in gene expression through the activation of NF-kB and STAT3. Because the expression of RAGE is under the control of NF-kB, RAGE activation results in RAGE upregulation through positive feedback. (**B**) In hypoxia, RAGE activation has been shown to stimulate HIF-1α through the activation of the PI3K/Akt and the RAS/RAF/MEK/ERK pathways. In hypoxia, nuclear HMGB1 translocates to the extracellular space through an unknown mechanism and interacts with RAGE. S100A4 and S100A8/A9 are upregulated and secreted in the tumor microenvironment and interact with RAGE. RAGE interaction with HMGB1, S100A4, and S100A8/A9 results in the activation of the MEK/ERK and PI3K/Akt pathways, resulting in changes in cancer-related gene expressions through the activation of NF-kB. NF-kB can further induce HIF-1a expression.

The three main signaling pathways activated in response to RAGE/ligand interactions are the MAPK/ERK, PI3K/Akt, and Jak/STAT pathways (Figure 3). Activation of the MAPK/ERK and PI3K/AKT pathways both lead to activation of NF-κB, resulting in the transcription of genes involved in cellular proliferation, apoptosis, and tumor growth [116,117,118,119,120,121,122,123]. In PC3 prostate cancer cells, activation of the PI3K/Akt pathway has been shown to regulate cell cycle progression through the phosphorylation of the retinoblastoma (Rb) protein, resulting in EF2-dependent gene transcription [124]. Similarly, in SiHa cervical squamous cancer cells, RAGE-dependent activation of the PI3K/Akt signaling pathway has been shown to promote cell cycle progression through the upregulation of the proliferating cell nuclear antigen (PCNA) [125].

In the JAK/STAT pathway, JAK phosphorylates STAT3 in response to IL-6, resulting in the translocation of STAT3 to the nucleus and enhanced cell proliferation [126,127,128,129]. Kang et al. reported that in pancreatic tumors, after its phosphorylation with JAK, STAT3 translocated to the mitochondria and increased ATP production, favoring tumor cell proliferation as a result of RAGE activation [128].

### 4.3. Hypoxia and RAGE Signaling in Cancer Tumors

The promoter region of RAGE contains at least one functional HRE (sequence: 5’-RCGTG-3’, where R is a purine base), and increased expression levels of RAGE have been observed during hypoxia in murine and rat models of brain and liver ischemic hypoxia [130,131,132,133,134,135]. These data suggest that RAGE is a potential target gene for both HIF-1a and HIF-2a since the HREs for both transcription factors (HIF-1a and HIF-2a) are identical [136]. RAGE signaling has also been associated with increased inflammation, as well as increased expression levels of RAGE ligands [131,132,137,138,139].

Few studies so far have investigated the role of RAGE in cancer tumors under hypoxic conditions (Figure 3) [140,141]. Two independent studies, the first one in breast and head and neck tumors, and the second one in pancreatic tumors, have shown that RAGE expression level was increased in these tumors under hypoxic conditions [140,141]. Tafani et al. reported that in breast and cervical cancer cell lines, the increase in RAGE expression levels was HIF-1α-dependent and was associated with the activation of ERK1/2, PI3K, as well as NF-κB, resulting in increased cell migration and invasion through the activation of MMPs [140]. These authors also showed that increases in HIF-1α resulted in direct activation of NF-κB as well as an indirect activation of NF-κB through the activation of RAGE [140].

Hypoxia has also been shown to modulate RAGE-dependent signaling pathways in pancreatic tumors [141]. Kang et al. showed that RAGE expression levels increased in hypoxic conditions, resulting in the activation of two different pathways: the KRAS dependent RAF/MEK/ERK pathway as well as the PI3K/Akt pathway [141]. Activation of both pathways was shown to lead to NF-κB activation as well as HIF-1α activation [141]. In this study, hypoxic conditions resulted in a RAGE-dependent activation of HIF-1α, but not in an HIF-1α dependent RAGE upregulation, as reported by Tafani et al. in breast and head and neck cancer tumors, suggesting distinct mechanisms of RAGE activity in hypoxia in different types of cancers [140]. Kang et al. further reported that under hypoxic conditions, RAGE activation was positively associated with tumor cell survival through increased autophagy and decreased apoptosis [141]

### 4.4. RAGE Ligands in Hypoxic Tumors

RAGE ligands consist of a large number of molecules structurally very diverse. RAGE ligands include advanced glycation end products (AGE) that provided the name to the receptor, S100 proteins, β-amyloid peptides, high mobility group box 1 (HMGB1) protein, transthyretin, the Mac-1 α2 integrin, and the C3a and C1q complement proteins [108,142,143,144]. We will only discuss here selected RAGE ligands that have been reported to be involved in the hypoxic response in tumors.

#### 4.4.1. HMGB1

HMGB1 has been found overexpressed in many types of tumors, including colon, colorectal, prostate, gastric, pancreatic, glioma, neuroblastoma, and melanoma tumors (reviewed in the work of [145]) [93,116,146,147,148,149,150,151]. HMGB1 has multiple functions depending on its cellular localization. In the nucleus, HMGB1 is a DNA binding protein and modulates DNA transcription and recombination [152,153,154,155]. HMGB1 interacts with DNA via two DNA binding regions called box A and box B [156,157]. Nuclear HMGB1 plays the role of DNA chaperone, regulates DNA damage repair, and contributes to the maintenance of genome stability [158]. Besides its function as a nuclear protein, HMGB1 has important cytoplasmic and extracellular functions [156,159,160,161]. Cytoplasmic HMGB1 plays a role in tumor progression by promoting autophagy and activating the inflammasome [162,163,164]. HMGB1 can also be secreted from cells either via an active process involving hyperacetylation and packaging of HMGB1 into secretory vesicles or via a passive process during cell necrosis [162]. Extracellular HMGB1 interacts with RAGE as well as with other cell surface receptors such as TLRs [160,165,166,167,168,169]. Activation of the HMGB1/RAGE axis has been shown to result in many cellular processes, including proliferation and migration, inflammation, and chemotaxis [170,171,172,173,174].

In hypoxia, melanoma cells have been shown to release higher levels of HMGB1 than in normoxia [150]. Huber et al. showed that HMGB1 released under hypoxia facilitated melanoma growth and metastasis, as well as tumor infiltration of tumor-promoting M2-like macrophages [150,175]. In addition, these macrophages were shown to synthesize IL-10 in a RAGE-dependent manner [150]. IL-10 is an inflammatory cytokine and has been shown to correlate with melanoma tumor progression and metastases formation [176]. In hepatocellular carcinoma (HCC) tumors, HMGB1 was shown to mediate tumor growth, under hypoxic conditions, through the interaction with intracellular TLR-9 [177] and to attract macrophages to the tumor site, resulting in increased metastasis [178]. In this study, however, the authors did not investigate if HMGB1 influenced RAGE signaling [177]. In a different study, Yan et al. showed that in HCC cells exposed to hypoxia, HMGB1 secreted extracellularly interacted with both TLR-4 and RAGE to promote cellular invasion through the activation of caspase 1, resulting in the cleavage and subsequent activation of the pro-inflammatory cytokines IL-1β and IL-18 [179]. HMGB1 is also a predictor of poor outcomes in glioblastoma patients [180]. Under hypoxic conditions, U118 glioblastoma cells were shown to release up to 50-fold higher HMGB1 levels than under normoxic conditions. HMGB1 containing condition media was shown to stimulate cell proliferation and invasion of these cells [180]. The mechanisms by which nuclear HMGB1 translocates to the extracellular milieu in hypoxic conditions are currently unknown.

#### 4.4.2. S100 Proteins

S100 proteins are small calcium-binding proteins that transmit calcium signals through their interaction with target proteins. S100 proteins exert both intracellular and extracellular functions [181,182]. Many S100 proteins interact with RAGE extracellularly and contribute to cancer cell proliferation, invasion, and metastasis [183,184]. Calcium has been shown to be released under hypoxic conditions and to stimulate HIF-1α expression and stabilization [185,186]. Changes in intracellular calcium levels in hypoxic cells could result in the activation of S100 dependent signaling pathways. S100 proteins could thus play critical functions during hypoxia. In this review, we will focus on six S100 members (S100B, S100P, S100A4, S100A7, S100A8, and S100A9) that have been investigated in the context of hypoxia in cancer.

##### 4.4.3. S100B

S100B is one of the best-characterized members of the S100 protein family [181,182]. S100B expression is highest in the brain, and high levels of S100B are associated with neurodegeneration and other neurologic disorders [187,188]. Interestingly, increased levels of S100B have been reported in the brain during ischemia [188], and S100B levels in umbilical cord blood have been suggested to be a potential biomarker of hypoxic-ischemic encephalopathy in asphyxiated newborns [189]. In human colon adenocarcinoma Caco-2 cells, activation of RAGE by extracellular S100B has been shown to upregulate HIF-1α through the activation of the p38/MAPK and Akt/mTOR signaling pathways, resulting in increased cell proliferation and angiogenesis [190]. This study was performed in normoxic conditions and showed that activation of the RAGE signaling pathways can regulate HIF-1α and the transcription of HIF-1α dependent genes.

##### 4.4.4. S100P

S100P was first isolated from the placenta and herein the letter P [191]. In tumors, S100P was found to promote tumor cell proliferation and survival, as well as metastasis through its interaction with RAGE [192,193,194,195]. A recent study showed that S100P played an important function during hypoxia by promoting cell migration and tumor metastasis in hepatocarcinoma cells and tumors [196]. In this study, the authors showed that under hypoxia, the levels of both caveolin 1 and S100P were increased, resulting in increased cell motility and metastasis, and that these effects were dependent on NF-κB [196]. Although the authors did not interrogate RAGE signaling pathways in their study, it is probable that the effect of S100P on cell migration, tumor growth, and metastasis was partially mediated by RAGE.

##### 4.4.5. S100A4

S100A4 is another ligand of RAGE that has been shown to stimulate tumor proliferation and metastasis in various cancer types through its interaction with RAGE [197,198,199,200,201]. In cancer tumors, S100A4 has been shown to increase cell motility, modulate cell proliferation and apoptosis, stimulate angiogenesis and trigger extracellular matrix protein remodeling (reviewed in the work of [199,200]).

In colorectal cancer (CRC), higher levels of HIF-1α were observed upon RAGE stimulation with human recombinant S100A4 [197]. Because HIF-1α activity in these tumors has been shown to enhance cellular motility and increased resistance to apoptosis and chemotherapy, it is reasonable to speculate that activation of the RAGE/S100A4 axis in CRC tumors favors tumor progression and chemoresistance through HIF-mediated mechanisms [202,203,204]. S100A4 expression levels are also regulated by hypoxia due to the presence of HRE in the gene coding S100A4 [205]. Hypoxic regulation of S100A4 was demonstrated in BGC823 gastric cancer cells where exposure to hypoxic conditions resulted in higher transcript levels of S100A4 as compared to normoxic conditions [205]. In a different study, Horiuchi et al. reported that in ovarian cancer cells, hypoxic-dependent expression of S100A4 was regulated by epigenetic mechanisms involving methylation of the HRE of S100A4 [206]. Increased levels of S100A4 transcripts and proteins were also observed in esophageal squamous cancer cells (ESCC) exposed to cobalt chloride, a hypoxia-mimetic agent that upregulates HIF-α [207,208].

##### 4.4.6. S100A7

S100A7 was initially named psoriasin because of its high expression level in psoriatic lesions [209]. Since its discovery, S100A7 has been shown to contribute to the progression of many cancers, including bladder, prostate, and skin cancers [210,211,212,213], and has been most studied in the context of breast cancer [12]. In breast cancer cells, intracellular S100A7 was shown to form a complex with the cofactor Jab1, resulting in the translocation of Jab1 to the nucleus and its interaction with HIF-1a, resulting in the transcription of HIF-1a dependent genes [214]. Interestingly, extracellular S100A7 has also been shown to stimulate hypoxic-dependent processes, such as angiogenesis, through its interaction with RAGE, in an insulin-like growth factor (IGF) dependent manner [215]. However, in this study, the authors did not investigate if the changes in angiogenesis w HIF dependent [215].

##### 4.4.7. S100A8/9

S100A8 and S100A9 are most commonly found as heterodimers [181,182,216]. Extracellular S100A8/A9 can bind to RAGE and other receptors described as S100 soil sensor receptors (SSSRs) [217], that include extracellular matrix metalloproteinase inducer (EMMPRIN), activated leukocyte cell adhesion molecule (ALCAM), TLR-4, neuroplastin β, and melanoma cells adhesion molecule (MCAM) [218,219]. In cancer, S100A8/A9 has been shown to have paradoxical functions by either promoting apoptosis or favoring tumor development, depending on the cellular context [220]. In tumors, activation of the S100A8/A9/RAGE pathway has been shown to promote tumor progression in several types of cancer, including breast, prostate, and colorectal cancer, through the activation of the MAPK pathway and NF-kB [178,221,222].

As reported with S100A4, hypoxia regulates S100A8/A9 expression through the presence of an HRE in the promoter region of both S100A8 and S100A9 genes, suggesting that these two proteins contribute to tumor progression in hypoxic conditions [223] (Table 1).

## 5. Conclusions

Hypoxia in tumors results in large cellular reprogramming. Recent studies have shown that activation of the RAGE signaling pathway could be a key player in cellular adaption to hypoxia. In this review, we discussed recent evidence on the role of RAGE and its ligands in stimulating tumor cell survival and favoring metastasis in hypoxic conditions. This emerging aspect of RAGE signaling in hypoxia deserves further attention, and inhibition of the RAGE signaling pathway could become a promising approach to reduce tumor growth in hypoxic regions of tumors.

## Figures and Tables

**Table 1:** Effect of hypoxia on RAGE ligand levels or RAGE/ligand signaling.

Ligands	Cancer Type	Pathological Response	Reference
HMGB1	Melanoma	Under hypoxia, melanoma cells release HMGB1, which promotes melanoma growth and metastasis	[150]
	Hepatocellular carcinoma	HMGB1 released under hypoxia promotes cellular invasion and metastasis	[177,179]
	Glioblastoma	HMGB1 released under hypoxia promotes cell proliferation and invasion	[180]
S100B	Colon adenocarcinoma	Stimulation of RAGE by S100B activates HIF1-α and the expression of HIF-1α dependent genes	[190]
S100P	Hepatocellular carcinoma	Hypoxia upregulates S100P and further promotes metastasis of HCC	[196]
S100A4	Colorectal Cancer	Activation of RAGE by S100A4 results in increased levels of HIF-1α	[197]
	Gastric, ovarian cancer	Hypoxia upregulates S100A4	[205]
S100A7	Breast Cancer	Activation of RAGE by S100A7 results in increased angiogenesis	[13]
S100A8/A9	Prostate Cancer	Hypoxia upregulates S100A8 and S100A9 transcript and protein levels	[223]

## Abbreviations

AGEs	Advanced glycation endproducts
ALCAM	Activated leukocyte cell adhesion molecule
ANGPTL4	Angiopoietin-like 4
ARNT1	Aryl hydrocarbon receptor nuclear translocator 1
bHLH	Basic-helix-loop-helix
CBP/p300	CREB binding protein/p300 coactivator
Cdc42	Cell division control protein 42
C-TAD	C-terminal transactivation domain
DAMP	Damage-associated molecular pattern
Dia-1	Diaphanous 1
EMMPRIN	Extracellular matrix metalloproteinase inducer
EMT	Epithelial mesenchymal transition
ERK	Extracellular-regulated kinase
HAF	Hypoxia-associated factor
HIF	Hypoxia-inducible factor
HIF-1α	Hypoxia-inducible Factor 1α
HMGB1	High mobility group box 1
HRE	Hypoxia-responsive elements
Ig	Immunoglobulin
Jak	Janus kinase
LZIP	Leucine zipper domain
MAP	Mitogen-activated protein
MCAM	Melanoma cells adhesion molecule
MDM2	Mouse double minute 2 homolog
MHC	Major histocompatibility complex
MMP	Matrix metalloproteinases
mTOR	Mechanistic target of rapamycin kinase
MyD88	Myeloid differentiation primary response 88
NF-κB	Nuclear factor kappa-light-chain-enhancer of activated B cells
N-TAD	N-terminal transactivation domain
NPTN	Neuroplastin β
OCT4	Octamer binding transcription factor 4
ODD	Oxygen-dependent degradation
PAS	Per-ARNT-Sim domains
PCNA	Proliferating cell nuclear antigen
PHD	Proline-hydroxylases
PI3K	Phosphoinositide 3-kinase
Rac 1	Ras-related C3 botulinum toxin substrate 1
RAGE	Receptor of advanced glycation end products
SRC-1	Nuclear receptor coactivator 1
SSSR	S100 soil sensor receptors
STAT	Signal transducer and activator of transcription
TIF2	Transcriptional mediator/intermediary factor 2
TIRAP	TIR domain-containing adaptor protein
TLR	Toll-like receptor
VHL	von Hippel-Lindau protein
VEGF	Vascular endothelial growth factor
